# 4-(4-Bromophenyl)-thiazol-2-amine derivatives: synthesis, biological activity and molecular docking study with ADME profile

**DOI:** 10.1186/s13065-019-0575-x

**Published:** 2019-04-23

**Authors:** Deepika Sharma, Sanjiv Kumar, Balasubramanian Narasimhan, Kalavathy Ramasamy, Siong Meng Lim, Syed Adnan Ali Shah, Vasudevan Mani

**Affiliations:** 1 Faculty of Pharmaceutical Sciences, Maharshi Dayanand University, Rohtak, 124001 India; 2 Faculty of Pharmacy, Universiti Teknologi MARA (UiTM), 42300 Bandar Puncak Alam, Selangor Darul Ehsan Malaysia; 3 Collaborative Drug Discovery Research (CDDR) Group, Pharmaceutical Life Sciences Community of Research, Universiti Teknologi MARA (UiTM), 40450 Shah Alam, Selangor Darul Ehsan Malaysia; 4 Atta-ur-Rahman Institute for Natural Products Discovery (AuRIns), Universiti Teknologi MARA, 42300 Bandar Puncak Alam, Selangor Darul Ehsan Malaysia; 5 Department of Pharmacology and Toxicology, College of Pharmacy, Qassim University, Buraidah, 51452 Kingdom of Saudi Arabia

**Keywords:** Synthesis, Molecular docking, Thiazole derivatives, Antimicrobial, Anticancer

## Abstract

In order to overcome the challenges of microbial resistance as well as to improve the effectiveness and selectivity of chemotherapeutic agents against cancer, a novel series of 4-(4-bromophenyl)-thiazol-2-amine derivatives was synthesized and its molecular structures were confirmed by physicochemical and spectral characteristics. The synthesized compounds were further evaluated for their in vitro antimicrobial activity using turbidimetric method and anticancer activity against oestrogen receptor positive human breast adenocarcinoma cancer cell line (MCF7) by Sulforhodamine B (SRB) assay. The antimicrobial activity results revealed that compound **p2**, **p3**, **p4** and **p6** exhibited promising antimicrobial activity that are comparable to standard norfloxacin (antibacterial) and fluconazole (antifungal). Anticancer screening results demonstrated that compound **p2** was found to be the most active one against cancer cell line when compared to the rest of the compounds and comparable to the standard drug (5-fluorouracil). The molecular docking study demonstrated that compounds, **p2**, **p3**, **p4** and **p6** displayed good docking score within binding pocket of the selected PDB ID (1JIJ, 4WMZ and 3ERT) and showed promising ADME properties.

**Electronic supplementary material:**

The online version of this article (10.1186/s13065-019-0575-x) contains supplementary material, which is available to authorized users.

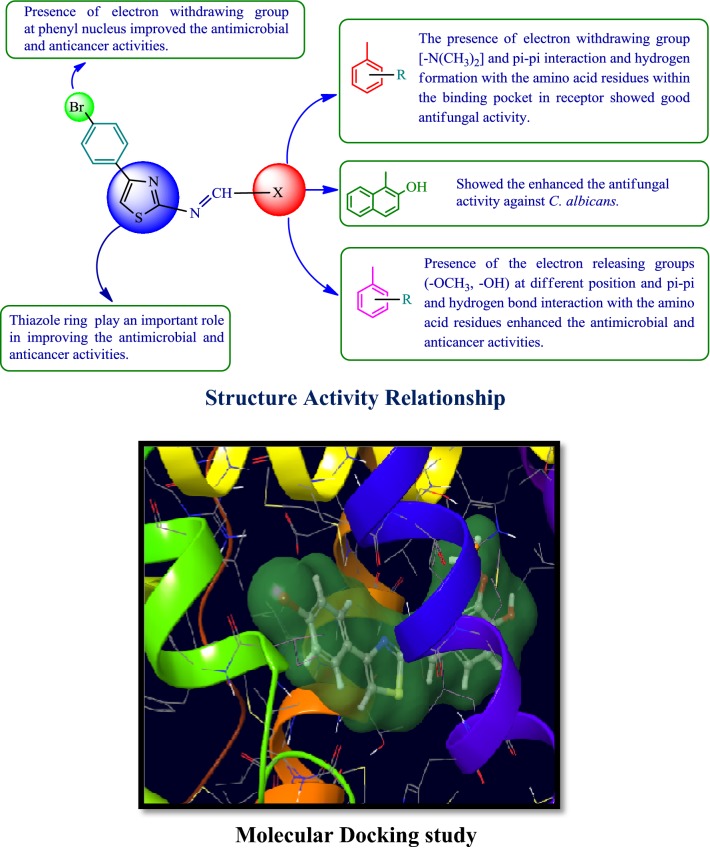

## Background

In recent years, epidemiological studies confirmed the significant negative impact of infections caused by pathogenic bacteria and fungi against human health. Large-scale surveillance revealed increasing incidence of drug-resistance that had compromised the efficacy of antimicrobial therapy. The increased emergence of multidrug-resistant pathogenic bacteria has called for exploration of alternative drug therapies [1]. As such, research is now focused towards new antimicrobial agents with expansion of bioactivity of existing drugs and also with novel target so as to address the problem of resistance [2].

In this era, cancer remains as one of the most serious clinical problems and the second primary cause of deaths worldwide. Cancer, which is characterized by uncontrollable division of abnormal cells, could be fatal if proliferation were to occur continuously [3]. Although many effective chemotherapeutic agents are available, they generally exhibit serious side-effects such as toxicity and resistance. With the increasing understanding of drugs’ cytotoxic mechanism of action and the discovery of specific target, novel chemical therapeutic drugs could be designed for treatment of cancer [4].

**Fig. 1:**
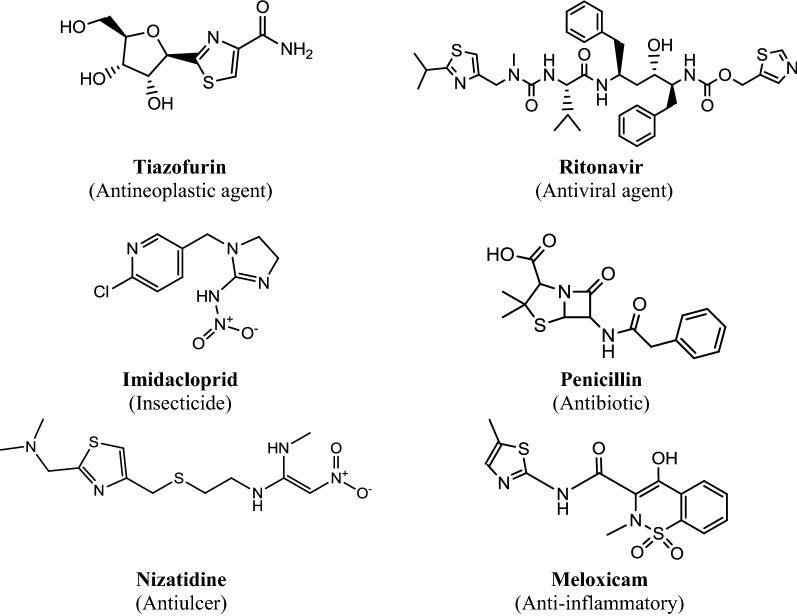
Marketed preparation of thiazole nucleus

It has been long since researchers show special interest in heterocyclic compounds that possess sulphur and nitrogen atom [5, 6]. Thiazole, for instance, exhibit widespread biological activities like antibacterial [7, 8], antimycobacterial [9], antileishmanial [10], anticancer [11] and antifungal [12]. The various marketed preparations that contain thiazole nucleus include tiazofurin (antineoplastic agent), ritonavir (antiviral agent), imidacloprid (insecticide), penicillin (antibiotic), nizatidin (antiulcer) and meloxicam (anti-inflammatory) (Fig. 1). The presence of electron withdrawing group at the *p*-position of phenyl nucleus directly attached to thiazole ring showed good antibacterial activity [8]. Schiff bases exerted various pharmacological activities such as anticancer, antimicrobial and antileishmanial amongst others [13, 14]. Aromatic substitution at *para* position of thiazole enhanced the anticancer activity. It can thus act as template for further investigation and synthesis of new derivatives [15].

Structure based drug designing (SBDD) and ligand based drug designing (LBDD) techniques are employed as important drug discovery tools in rational drug designing process [16]. Molecular docking is the advanced computational used techniques in SBDD to obtain optimized conformation of ligand–receptor interaction and to study their relative orientation through the minimized energy free system [17]. Computer aided drug designing (CADD) is fast, economical modernized technique that gives valuable, accurate and deep understandings of experimental findings and new suggestions for molecular structures to be synthesized [18].

Drug molecules might fail during development because of several reasons but as found by the researchers one of the major reasons of failures is related with poor pharmacokinetic and absorption, distribution, metabolism and excretion (ADME) properties [19]. Unexpected drug toxicity is the one of the major factors to withdraw drug from the market. Therefore, ADME properties are the crucial determinants for the clinical success of the drug [20].

Lipinski’s rule of five is a rule of thumb to evaluate druglikeness or determine if a chemical compound with a certain pharmacological or biological activity has chemical properties and physical properties that would make it a likely orally active drug in humans. The rule describes molecular properties important for a drug’s pharmacokinetics in the human body, including their absorption, distribution, metabolism, and excretion (ADME). The rule is important to keep in mind during drug discovery when a pharmacologically active lead structure is optimized step-wise to increase the activity and selectivity of the compound as well as to ensure drug-like physicochemical properties are maintained as described by Lipinski’s rule which states that (i) no more than 5 hydrogen bond donors (ii) no more than 10 hydrogen bond acceptors (iii) a molecular mass less than 500 Da (iv) an octanol–water partition coefficient log *P* not greater than 5 (https://en.wikipedia.org/wiki/Lipinski%27s_rule_of_five).

**Fig. 2:**
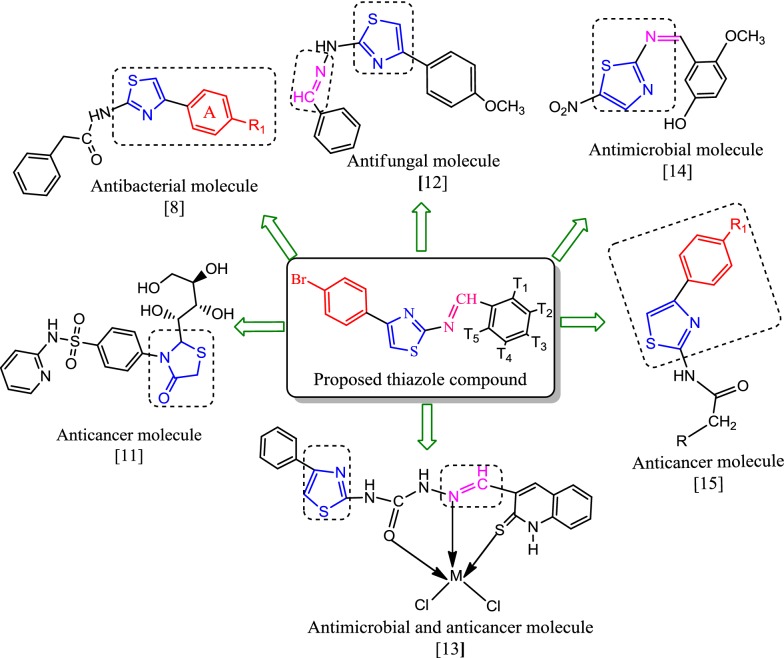
Design of proposed thiazole molecules for antimicrobial and anticancer potential based on literature

Now these days, computational approaches are employed to determine the ADME of the drug molecules. ADME modeling has attracted the considerable attention of the pharmaceutical researchers for the drug discovery as they are high-throughput in nature and cost effective [21]. As a part of our continuous efforts in finding new antimicrobial and anticancer [22, 23] mentioned above, in the present study, 4-(4-bromophenyl)-thiazol-2-amine derivatives were designed for assessment of their antimicrobial and antiproliferative potentials (Fig. 2).

## Results and discussion

### Chemistry

**Scheme 1:**
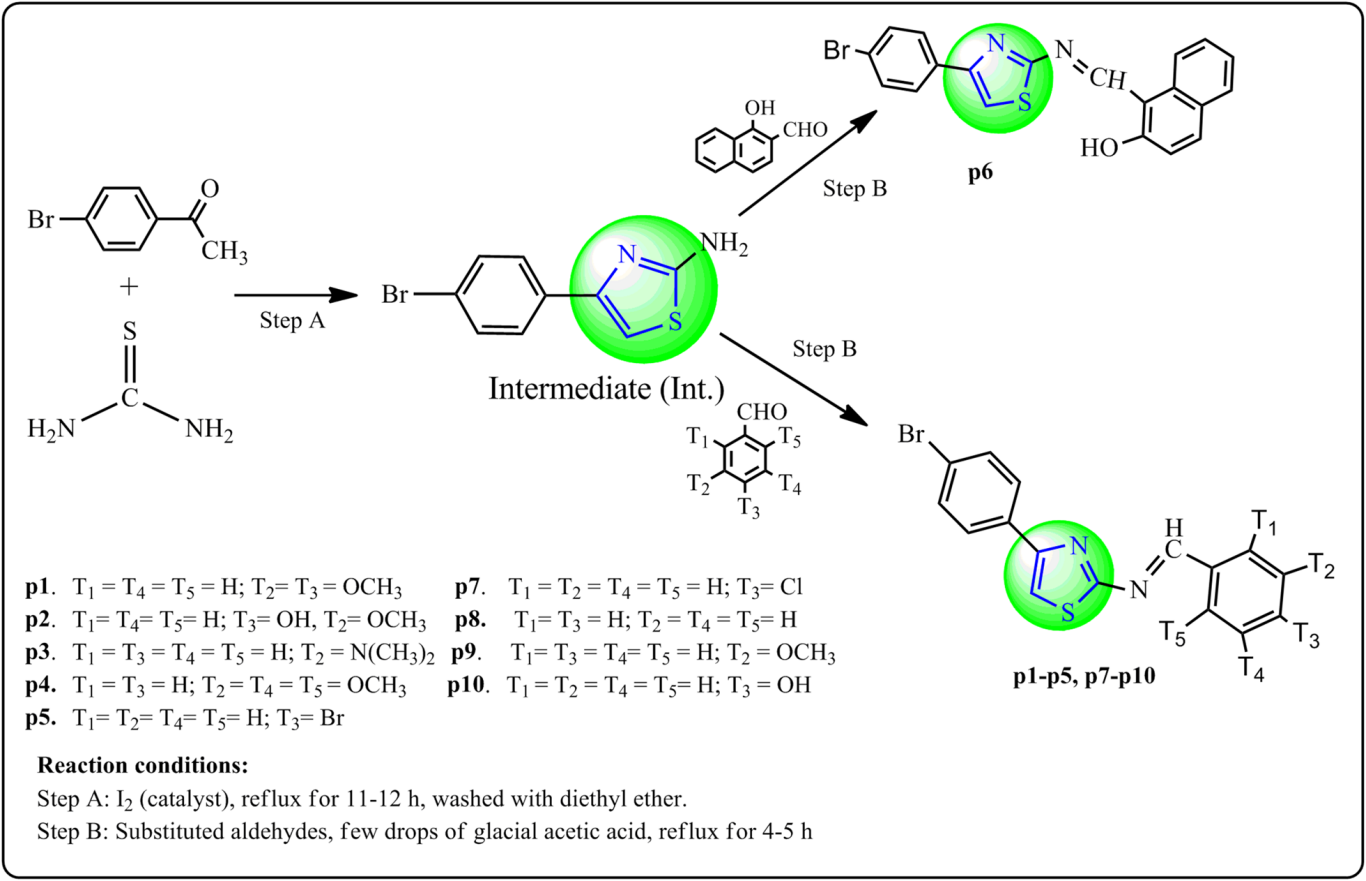
For the synthesis of 4-(4-bromophenyl)thiazol-2-amine derivatives (**p1**–**p10**)

Synthesis of intermediate and target derivatives (**p1**–**p10**) were carried out as per the reactions outlined in Scheme 1. Initially, *p*-bromoacetophenone and thiourea were reacted in the presence of catalyst iodine to yield the 4-(4-bromophenyl) thiazol-2-amine (Intermediate). The intermediate with corresponding aromatic aldehyde yielded the target compounds (**p1**–**p10**). The molecular structures of the synthesized compounds were confirmed by physicochemical properties (Table 1) and spectral characteristics (Table 2). IR spectrum of intermediate showed the characteristic IR band at 817 cm^−1^ and 666 cm^−1^ which indicated the presence of N–H str. of NH_2_ and C–Br str. of C_6_H_5_Br, respectively. The presence of stretch at 1265 cm^−1^ displayed the C–N connectivity and showed the presence of Ar–NH_2_ linkage. The IR stretch present at 725 cm^−1^ and 1632 cm^−1^ showed the C–S and C=N linkage, respectively, therefore these all linkages indicates the existence of thiazole nucleus within the structure of the (Intermediate). The occurrence of band at 3113 cm^−1^ and 1586 cm^−1^ indicated the presence of C–H skeletal and C=C skeletal structure, respectively within the phenyl nucleus. The molecular structures of synthesized compounds were further confirmed by ^1^H NMR spectral data. The ^1^H-NMR spectrum of intermediate showed singlet at 6.9 δ ppm showed the presence of NH_2_ group. The ^1^HNMR spectra of synthesized derivatives displayed multiplet at 6.939–7.52 δ ppm due to presence of aromatic C–H linkage. The presence of singlet at 7.57–9.7 δ ppm displayed the N=CH connectivity, therefore the confirmation of the presence of benzylidene linkage within the synthesized derivatives. The presence of O–CH_3_ of Ar–OCH_3_ was confirmed by the appearance of singlet at 3.76–3.9 δ ppm. All compounds showed singlet at 6.9–7.80 δ ppm due to the existence of C–H in thiazole ring. Compound **p2** showed singlet at 5.39 δ ppm due to presence of –OH at the *para* position. Compound **p3** showed singlet at 2.91 δ ppm due to presence of –N(CH_3_)_2_ at the *para* position. ^13^C-NMR spectra of the thiazole derivatives was displayed the fine conformity of their proposed molecular structure i.e. the carbon atoms of phenyl nucleus found around 120.4, 109.4, 128.7, 122.2, 110.3, 150.8 δ ppm, carbon atoms of thiazole around 150.3, 172.4, 109.6 and carbon of N=CH group at 159.4 δ ppm. Elemental analysis results of the thiazole derivatives were lie within the limits of ± 0.5% of the theoretical results.

**Table 1:** Physicochemical properties of the synthesized compounds (p1–p10)

S. no	Compound		Molecular formula	Color	m.pt. °C	R_*f*_ value	% Yield
1	**(p1)**	4-(4-Bromophenyl)-*N*-(3,4-dimethoxybenzylidene)thiazol-2-amine	C_18_H_15_N_2_SBrO_2_	Light yellow	134–136	0.45	85
2	**(p2)**	4-((4-(4-Bromophenyl)thiazol-2-ylimino)methyl)-2-methoxyphenol	C_17_H_13_N_2_SBrO_2_	Creamy yellow	122–125	0.38	70
3	(**p3)**	4-(4-Bromophenyl)-*N*-(4-(dimethylamino)benzylidene)thiazol-2-amine	C_18_H_16_N_3_SBr	Yellowish white	117–120	0.25	65
4	**(p4)**	*N*-(3,4,5-Trimethoxybenzylidene)-4-(4-bromophenyl)thiazol-2-amine	C_19_H_17_N_2_SBrO_3_	Greenish yellow	105–107	0.30	75
5	**(p5)**	*N*-(4-Bromobenzylidene)-4-(4-bromophenyl)thiazol-2-amine	C_16_H_10_N_2_SBr_2_	Dark yellow	62–64	0.38	69
6	**(p6)**	1-((4-(4-Bromophenyl)thiazol-2-ylimino)methyl)naphthalen-2-ol	C_20_H_13_BrN_2_OS	Light yellow	105–107	0.30	72
7	**(p7)**	*N*-(4-Chlorobenzylidene)-4-(4-bromophenyl)thiazol-2-amine	C_16_H_10_BrClN_2_S	Light yellow	65–70	0.23	83
8	**(p8)**	*N*-(2,4-Dimethoxybenzylidene)-4-(4-bromophenyl)thiazol-2-amine	C_18_H_15_BrN_2_O_2_S	Lemon yellow	71–73	0.52	72
9	**(p9)**	*N*-(3-Methoxybenzylidene)-4-(4-bromophenyl)thiazol-2-amine	C_17_H_13_BrN_2_OS	Yellowish white	188–190	0.79	65
10	**(p10)**	3-(((4-(4-Bromophenyl)thiazol-2-yl)imino)methyl)phenol	C_16_H_11_BrN_2_OS	Dark yellow	106–108	0.21	71

**Table 2:** Spectral data of the synthesized compounds and intermediate (Int.)

Sr. no.	IR KBr (cm^−1^)						^1^H-NMR (DMSO-d_6_, ppm)	^13^C-NMR (DMSO-d_6_, ppm)	MS: *m/z* (M^+^+1)	Elemental analysis (CHN)
	C=C str.	C–Br str.	C–O–C str. OCH_3_	C–S str.	C=N str.	Other (str./bending)				
Int.	1586	666	–	725	1632	817 (N–H str., NH_2_), 1265 (C–N str., Ar–NH_2_)	7.19–7.56 (m, 4H, ArH), 6.90 (s, 1H, CH of thiazole), 4.12 (s, 2H, –NH_2_ of thiazole)	127.4, 131.1, 122.6, 130.2, 127.3, 130.8 (6C, phenyl nucleus), 167.7, 100.4, 151.5 (3C, thiazole)	255	Theoretical calc: C, 42.37; H, 2.77; N, 10.98; Found: C, 42.31; H, 2.74; N, 10.89
**p1**	1587	642	1266	725	1674	–	6.93 (m, 7H, aromatic H), 7.42 (s, 1H, CH of thiazole)	120.4, 109.4, 128.7, 122.2, 110.3, 150.87, 123.2, 110.0, 150.2, 110.6, 148.5, 128.6 (12C, phenyl nucleus), 150.3, 172.41, 109.6 (3C, thiazole), 159.4 (N=CH), 55.2, 54.6 (2C, OCH_3_)	404	Theoretical calc: C, 53.61; H, 3.75; N, 6.95; Found: C, 53.48; H, 3.69; N, 6.81
**p2**	1590	601	1208	756	1673	–	6.76–7.45 (m, 7H, ArH), 7.43 (s, 1H, C–H of thiazole), 8.85 (s, 1H, N=CH), 3.71 (s, 3H, OCH_3_), 5.39 (s, 1H, OH)	130.4, 127.4, 124.7, 132.1, 130.3, 131.8, 119.8, 115.4, 150.2, 147.6, 113.4, 127.9, (12C, phenyl nucleus), 151.3, 173.1, 108.6 (3C, thiazole), 158.2 (N=CH), 55.7 (O–CH_3_), 53.4 (1C, –OCH_3_)	390	Theoretical calc: C, 52.45; H, 3.37; N, 7.20; Found: C, 52.33; H, 3.41; N, 7.18
**p3**	1588	726	1229	812	1658	830 [(C–N str., N(CH_3_)_2_]	6.80–7.41 (m, 8H, ArH), 7.80 (s, 1H, –N=CH), 3.43 (s, 3H, OCH_3_), 7.45 (s, 1H, CH of thiazole)	130.4, 127.4, 124.7, 132.1, 130.3, 131.8, 124.6, 129.1, 110.8, 152.2, 110.8, 127.3 (12C, phenyl nucleus), 151.3, 173.41, 108.6 (3C, thiazole), 158.2 (N=CH), 40.8, 39.9 (2C, N(CH_3_)_2_)	375	Theoretical calc: C, 57.00; H, 4.53; N, 10.50; Found: C, 57.04; H, 4.42; N, 10.47
**p4**	1588	575	1232	705	1682	1325 (C–O str. and O–H in plane bending, phenol)	6.9–7.39 (m, 6H, ArH), 7.73 (s, 1H, –N=CH), 3.73 (s, 3H, OCH_3_), 7.47 (s, 1H, CH of thiazole), 2.91 (s, 6H, N(CH_3_)_2_)	121.5, 107.2, 128.7, 123.1, 111.1, 151.2, 151.1, 102.3, 130.1, 103.1, 152.1, 140.5 (12C, phenyl nucleus), 149.3, 171.2, 109.8 (3C, thiazole), 160.1 (N=CH), 59.6, 55.8 (2C, OCH_3_, 54.3, 58.5 (2C, OCH_3_)	422	Theoretical calc: C, 52.66; H, 3.95; N, 6.46; Found: C, 52.54; H, 3.87; N, 6.44
**p5**	1587	589	–	829	1677	–	7.28–7.60 (m, 8H, ArH), 7.90 (s, 1H, N=CH), 7.49 (s, 1H, CH of thiazole)	120.1, 126.7, 123.1, 111.1, 151.2, 109.2, 130.1, 126.2, 131.2, 122.8, 129.7, 128.6, 131.2 (12C, phenyl nucleus), 149.3, 171.2, 109.8 (3C, thiazole), 160.1 (N=CH), 59.6 (C, –OCH_3_)	423	Theoretical calc: C, 45.52; H, 2.39; N, 6.64; Found: C, 45.48; H, 2.27; N, 6.51
**p6**	1590	592	–	742	1618	1395 (C–O str. and O–H in plane bending, phenol),	7.36–8.01 (m, 10H, ArH), 8.14 (s, 1H, N=CH), 7.43 (s, 1H, CH of thiazole)	122.6, 126.7, 132.1, 118.1, 151.2, 128.21, 131.4, 119.5, 170.5, 107.9 (12C, naphthalene nucleus), 148.3, 169.1, 109.1 (3C, thiazole), 159.10 (N=CH)	410	Theoretical calc: C, 58.69; H, 3.20; N, 6.84; Found: C, 58.55; H, 3.18; N, 6.83
**p7**	1590	607	–	829	1521	725 (C–Cl str., ArCl)	7.24–7.58 (m, 8H, ArH), 7.90 (s, 1H, N=CH), 7.43 (s, 1H, CH of thiazole)	127.2, 129.4, 134.5, 129.8, 135.6, 126.3, 129.8, 133.2, 131.4, 124.8, 135.5 (12C, phenyl nucleus), 149.5, 173.2, 108.8 (3C, thiazole), 159.1 (N=CH)	380	Theoretical calc: C, 50.88; H, 2.67; N, 7.4; Found: C, 50.76; H, 2.51; N, 7.2
**p8**	1583	572	1263	720	1675	–	6.80–7.35 (m, 7H, ArH), 7.90 (s, 1H, N=CH), 7.36 (s, 1H, CH of thiazole), 3.88 (s, 1H, OCH_3_)	124.2, 134.5, 130.2, 133.5, 128.8, 104.5, 132.1, 115.1, 158.3, 100.2, 161.8 (12 C, phenyl nucleus), 149.5, 173.2, 108.8 (3C, thiazole), 161.2 (N=CH), 54.6, 53.5 (2C, OCH_3_)	404	Theoretical calc: C, 53.61; H, 3.75; N, 6.95; Found: C, 53.59; H, 3.64; N, 6.81
**P9**	1532	659	1265	805	1583	–	6.90–7.27 (m, 8H, ArH), 7.40 (s, 1H, N=CH), 7.38 (s, 1H, CH of thiazole), 3.90 (s, 1H, OCH_3_)	125.2, 133.5, 131.2, 127.5, 126.8, 127.6, 120.1, 136.5, 110.1, 159.7 (12C, phenyl nucleus), 151.5, 172.3, 107.6 (3C, thiazole), 160.2 (N = CH), 54.3 (O-CH_3_)	374	Theoretical calc: C, 54.70, H, 3.51; N, 7.50; Found: C, 54.56; H, 3.28; N, 7.38
**p10**	1505	720	1265	829	1583	1397 (C–O str. and O–H in plane bending, phenol)	6.70–7.38 (m, 8H, ArH), 9.70 (s, 1H, N=CH), 7.52 (s, 1H, CH of thiazole)	127.2, 132.5, 130.2, 126.5, 125.8, 115.2, 157.3, 112.2, 136.5, 120.0, 131.1 (12C, phenyl nucleus), 150.5, 171.3, 108.5 (3C, thiazole), 160.1 (N=CH)	360	Theoretical calc: C, 53.49; H, 3.09; N, 7.80; Found: C, 53.41; H, 3.07; N, 7.72

### In vitro antimicrobial activity

**Fig. 3:**
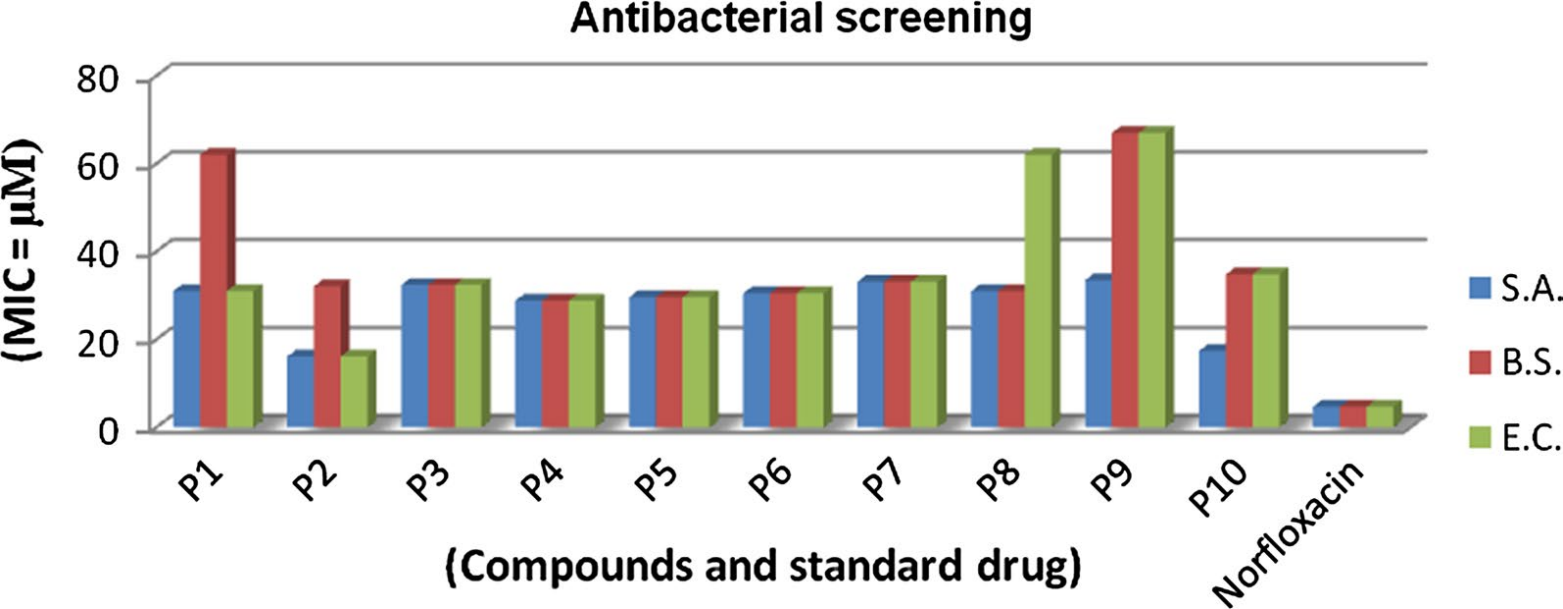
Graphical representation of antibacterial activity of synthesized compounds

**Fig. 4:**
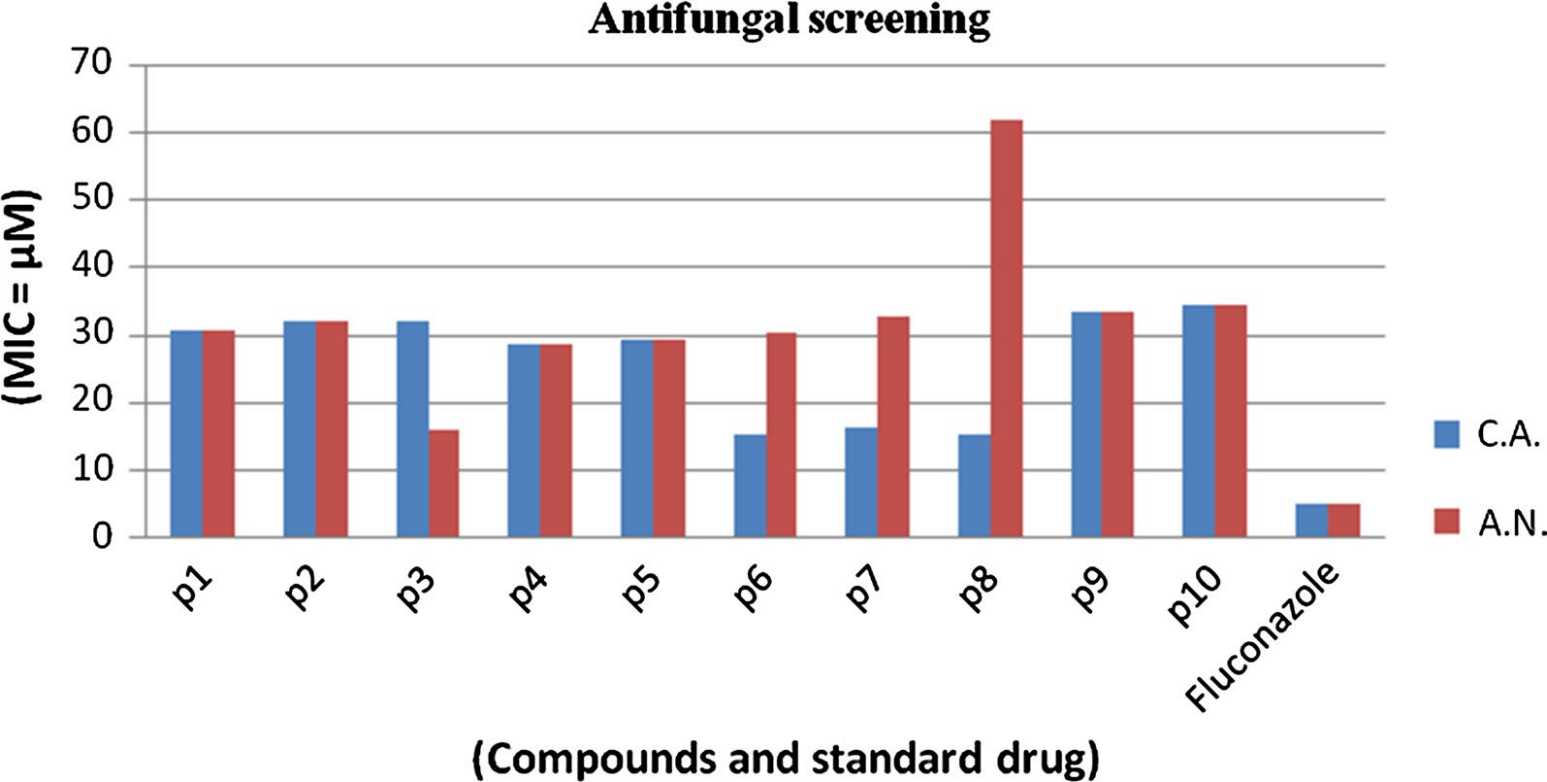
Graphical representation of antifungal activity of synthesized compounds

The antimicrobial potential of synthesized molecules was determined using turbidimetric (tube dilution method). The antibacterial activity was determined against Gram-negative bacterium: *Escherichia coli* (MTCC443) and Gram-positive bacteria: *Staphylococcus aureus* (MTCC3160), *Bacillus subtilis* (MTCC441) and compared to positive control norfloxacin. The antifungal study of compounds was carried out against fungal strains: *Candida albicans* (MTCC227) and *Aspergillus niger* (MTCC281) and compared to positive control (fluconazole). The results of antibacterial and antifungal evaluation were recorded in terms of minimum inhibitory concentration (MIC) (Table 3, Figs. 3 and 4).

**Table 3:** Antimicrobial and anticancer screening results of synthesized thiazole molecules (p1–p10)

Compound	(Antimicrobial screening) MIC = µM					^a^IC_50_ = µM
	Microbial species					
	Bacterial			Fungal		Cancer cell line
	*S.A.*	*B.S.*	*E.C.*	*C.A.*	*A.N.*	MCF7
**p1**	31	62.0	31	31	31	17.4
**p2**	16.1	32.1	16.1	32.1	32.1	10.5
**p3**	32.4	32.4	32.4	32.4	16.2	37.4
**p4**	28.8	28.8	28.8	28.8	28.8	38.0
**p5**	29.6	29.6	29.6	29.6	29.6	40.3
**p6**	30.5	30.5	30.5	15.3	30.5	73.3
**p7**	33.1	33.1	33.1	16.5	33.1	47.6
**p8**	31	31.0	62.0	15.5	62.0	52.1
**p9**	33.5	67.0	67.0	33.5	33.5	17.2
**p10**	17.4	34.8	34.8	34.8	34.8	21.4
Norfloxacin	4.7	4.7	4.7	–	–	–
Fluconazole	–	–	–	5.0	5.0	–
5-Fluorouracil	–	–	–	–	–	5.2

*S.A.*: *Staphylococcus aureus* (MTCC3160); *B.S.*: *Bacillus subtilis* (MTCC441); *E.C.*: *Escherichia coli* (MTCC443); *C.A.*: *Candida albicans* (MTCC227) and *A.N.*: *Aspergillus niger* (MTCC281)

^a^IC_50_ is the concentration required to inhibit 50% of cell growth

In vitro antimicrobial results of developed compounds **p2** (MIC_sa_ and MIC_ec_ = 16.1 µM) showed promising potential against *S. aureus* and *E. coli*, respectively. Compound **p4** (MIC_bs_ = 28.8 µM) displayed potent antibacterial activity against *B. subtilis*. Antifungal activity results demonstrated that compound **p6** (MIC_ca_ = 15.3 µM) displayed significant antifungal activity against *C. albicans* and compound **p3** (MIC_an_ = 16.2 µM) was found to be most potent against *A. niger.*

### In vitro anticancer activity

**Fig. 5:**
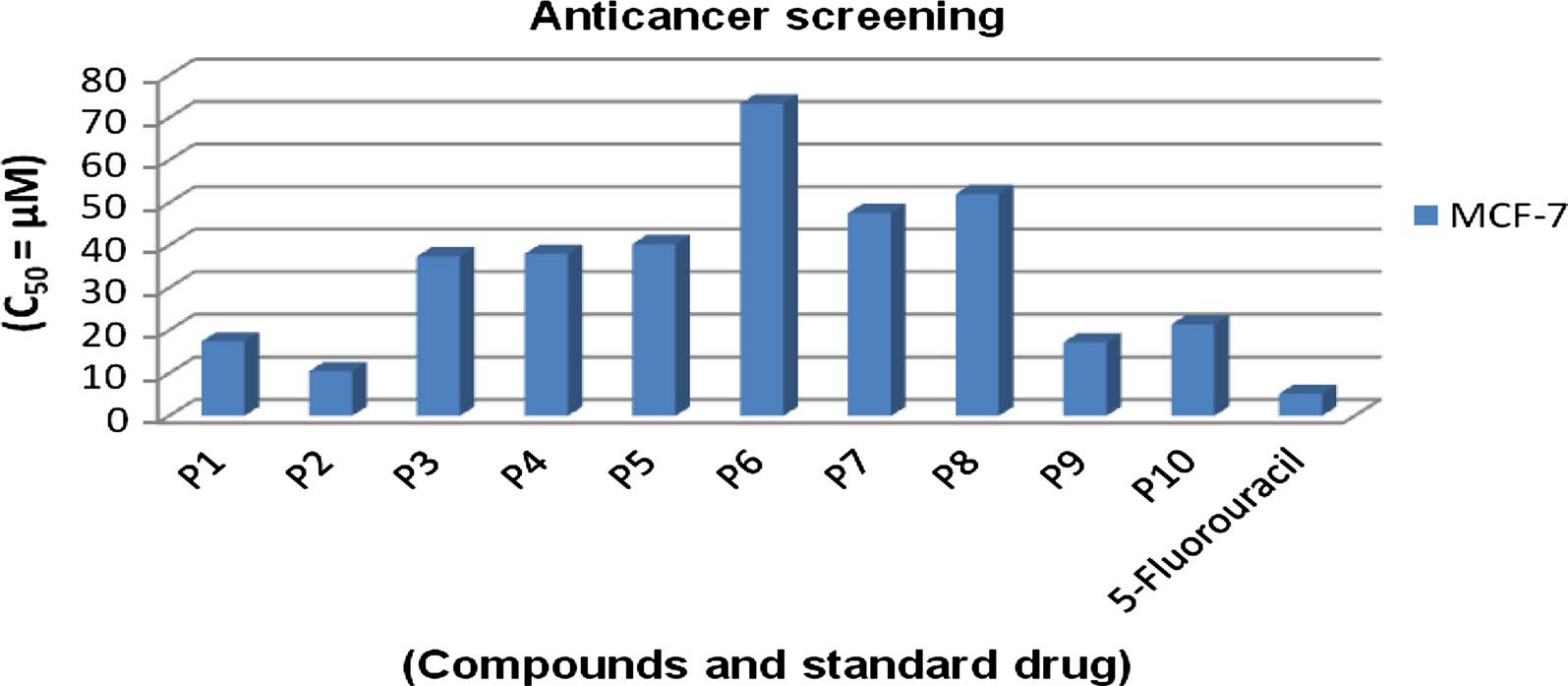
Graphical representation of anticancer activity of synthesized compounds

Anticancer activity of the synthesized thiazole compounds was tested against an oestrogen receptor positive human breast adenocarcinoma cell line (MCF7) using the SRB colorimetric assay in comparison to a standard drug (5-fluorouracil). Anticancer activity results (Table 3 and Fig. 5) revealed that thiazole exhibited good anticancer potential against cancer cell line MCF7. Compound **p2** (IC_50_ = 10.5 μM), in particular, exhibited anticancer activity and almost comparable to the reference drug, 5-fluorouracil (IC_50_ = 5.2 μM).

### Molecular docking

**Fig. 6:**
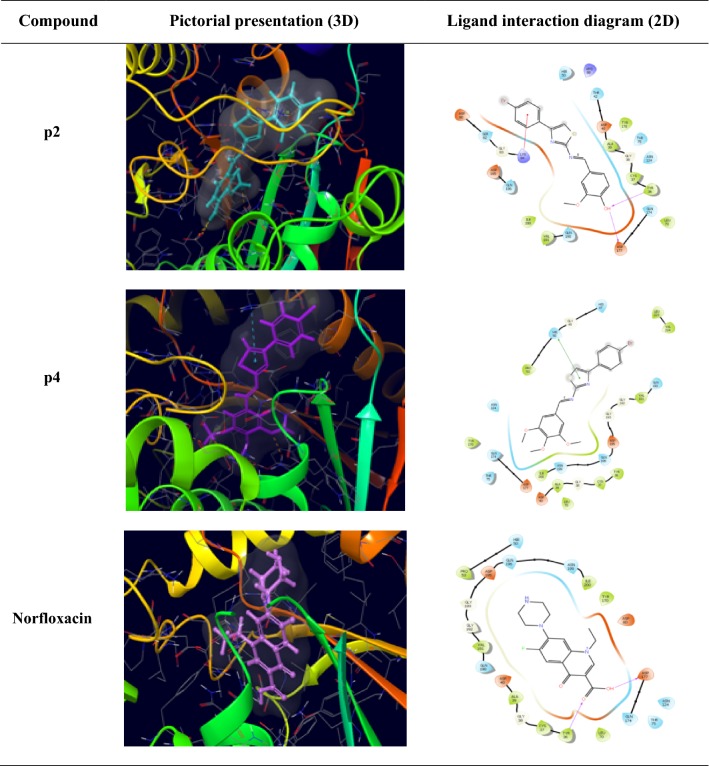
Pictorial presentation (3D) and Ligand interaction diagram (2D) of most active antibacterial compounds (**p2**, **p3** and **p4**) and standard norfloxacin

Molecular docking is done to study the binding mode of the synthesized 4-(4-bromophenyl)-thiazol-2-amine derivatives with their respective receptors. The PDB files required were identified through literature survey. Docking studies of the most active compounds were carried out using GLIDE module of docking software Schrodinger *v11.5*. Docking score values were used to rank the conformations of these ligand–receptor complexes. Molecular docking study of the most active antibacterial compounds **(p2** and **p4)** and standard drug (norfloxacin) was done in the active sites of topoisomerase II (PDB ID: 1JIJ) obtained from the protein data bank. The ligand interaction diagram (2D) and pictorial presentation (3D) of docked compound and standard drug are shown in Fig. 6. The 2D ligand interaction diagrammatic view depicted that these compounds share same homology with standard norfloxacin (Table 4) by interacting with similar amino acid residues. The compound **p2** form H-bond with amino acids Tyr36 and Asp177 that is responsible for good antibacterial activity.

**Table 4:** Docking results of most active (antibacterial and antifungal) compounds and standard drugs

Compound	Docking score	Glide energy (kcal/mol)	Interacting amino acid residues
**p2**	− 5.547	− 49.479	Asp177, Gln174, Tyr36, Cys37, Ala39, Asp40, Thr42, Lys84, Gly83, Ser82, Asp80
**p3**	− 6.513	− 48.914	His468, Cys470, Phe463, Leu380, Pro379, Hie378, Thr322, Ser319, Tyr126
**p4**	− 4.845	− 54.654	His47, Gly49, Hie50, Pro53, Gln174, Asp177, Ile200, Asn199, Gln196, Asp195, Gly193, Gly192
**p6**	− 8.342	− 45.842	Tyr126, Leu129, Thr130, Tyr140, Ile139, Cys470, Thr322, Ser319, Thr318
**Norfloxacin**	− 6.18	− 53.349	Asp177, Glu174, Leu70, Tyr36, Cys37, Gly38, Ala39, Asp40, Gln190, Val191, Gly192
**Fluconazole**	− 5.847	− 40.932	His468, Arg469, Cys470, Ile471, Tyr126, Hie378, Leu380, Leu383, Arg385

**Fig. 7:**
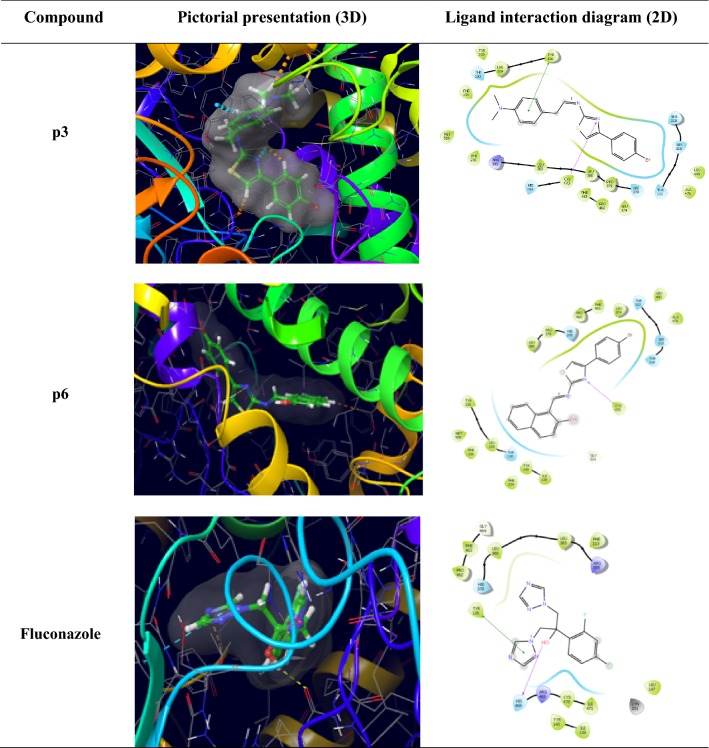
Pictorial presentation (3D) and ligand interaction diagram (2D) of most active antifungal compounds (**p3** and **p6**) and standard fluconazole

**Fig. 8:**
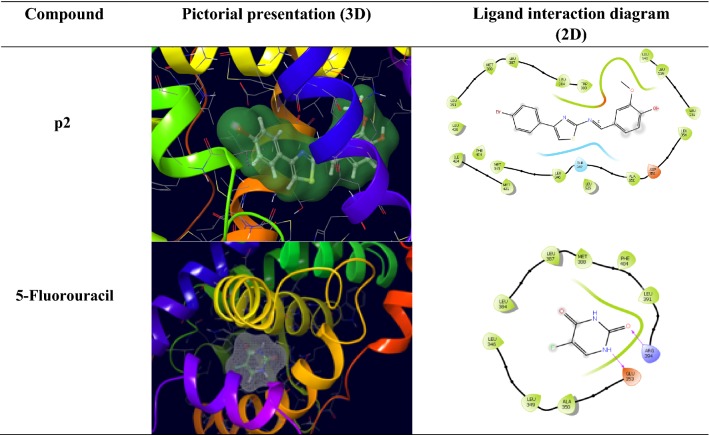
Pictorial presentation (3D) and ligand interaction diagram (2D) of most active compound (**p2**) and standard 5-fluorouracil

Molecular docking study of the most active antifungal compound **p3**, **p6** and standard drug (fluconazole) was done against active sites of lanosterol alpha demethylase (PDB: 4WMZ) obtained from the protein data bank. The ligand interaction diagram (2D) and pictorial presentation (3D) of docked compound and standard drug are as shown in Table 4 and Fig. 7. The diagrammatic view depicted that this compound share similar homology with that of standard fluconazole. The nitrogen atom of thiazole nucleus of compound **p3** and **p6** form H-bond with Cys470 amino acid residue. The compound **p3** also show pi–pi interaction with Tyr126 amino acid residue. The most active anticancer compound **p2** was docked in the binding pocket of ER-alpha of MCF7 (PDB ID-3ERT) co-crystallized with tamoxifen ligand. The results were examined based on docking score obtained by molecular docking software (Fig. 8, Table 5). The docking score was illustrated in the negative terms. More negative the docking score better would be the binding affinity of ligand with the receptor.

**Table 5:** Docking results of most active anticancer compound p2 with standard drug

Compound	Docking score	Glide energy (kcal/mol)	Interactive residues
**p2**	− 6.732	− 42.44	Trp383, Leu384, Leu387, Met388, Leu391, Phe404, Met343, Leu346, Met347
**5-Fluorouracil**	− 3.414	− 24.58	Glu353, Ala350, Leu349, Leu346, Leu348, Leu387, Met388, Phe404, Leu391, Arg394

### ADME results

Determination of ADME parameters of the synthesized 4-(4-bromophenyl)-thiazol-2-amine derivatives were done using QikProp module of Schrodinger *v11.5*. Around five physically relevant and pharmacologically significant parameters of the most active compounds, **p2**, **p3**, **p4** and **p6** were determined and summarized in Table 6. The compound **p2** lie within the range of all the parameters of Lipinski rule of five while rest of the potent compounds followed the Lipinski rule of five except the lipophilicity parameter and these compounds can be further optimized to improve their lipophilicity. The results displayed that compounds, **p2**, **p3**, **p4** and **p6** lie within the close agreement with the Lipinski’s rule thus making these derivatives as useful lead molecules for further study.

**Table 6:** ADME parameters of most active antimicrobial and anticancer compounds

Comp.	Molecular structure	ADME parameters					
		Mol MW	QPlogPo/w	DonorHB	AccptHB	Percent human oral absorption	Rule of five
**p2**		389.266	4.607	1.0	4.0	100.0	0
**p3**		400.335	5.71	0.0	3.5	100.0	1
**p4**		433.319	5.447	0.0	4.75	100.0	1
**p6**		409.299	5.52	1.0	3.25	100.0	1

### Structure activity relationship (SAR) studies

**Fig. 9:**
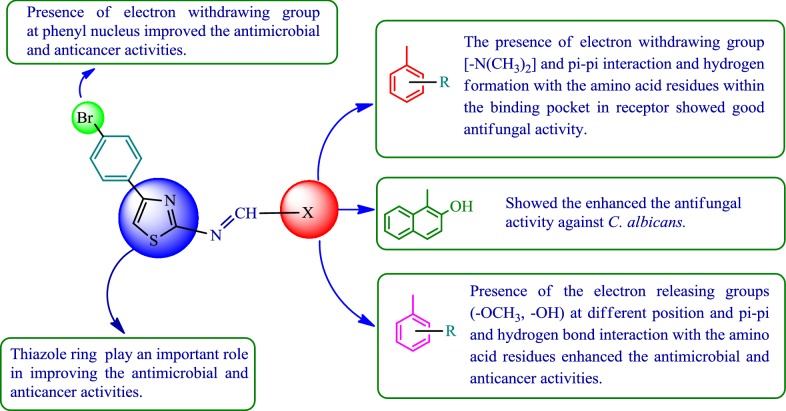
Structural activity relationship of synthesized thiazole derivatives

The in vitro antimicrobial and cytotoxicity outcomes demonstrated the following structure activity relationship for 4-(4-bromophenyl)-thiazol-2-amine derivatives (Fig. 9):

- Electron withdrawing group (Br) present at *para*-position of phenyl nucleus directly attached to thiazole ring improved antimicrobial and anticancer activities of 4-(4-bromophenyl)-thiazol-2-amines.
- The presence of electron withdrawing group [–N(CH_3_)_2_] on phenyl nucleus of synthesized compound **p3** and the pi–pi interaction with the amino acid residues in the binding pocket in receptor, enhanced antifungal activity. Compound **p6** (having 2-OH naphthaldehyde), improved antifungal activity against *C. albicans.*
- The presence of electron releasing group [–OCH_3_] on benzylidene portion of synthesized molecules (compound **p4**) and the pi–pi interaction with the amino acid residue within the binding pocket produce moderate antimicrobial activity.
- The presence of electron releasing groups [OH, –OCH_3_] on benzylidene portion of synthesized molecules (compound **p2**) and the pi–pi and hydrogen bond interaction with the amino acid residues enhanced antimicrobial and anticancer activities.

From result of structure activity relationship study, we may conclude that different structural requirements are required for a molecule to be effective against different goal. The aforementioned facts are supported by the earlier research findings [8, 24, 25].

## Experimental section

### Materials and methods

Preparatory material required for carrying out the research work was obtained from the commercial sources [Loba Chemie, Pvt Ltd. Mumbai, India Central Drug House (CDH) Pvt. Ltd., New Delhi, India] and used without further purification. The purity of the synthesized compounds was observed by thin layer chromatography (commercial silica gel plates (Merck), Silica gel F254 on aluminium sheets) using chloroform:toluene (7:3, v/v) as mobile phase. Sonar melting point apparatus was used to determine the melting point of synthesized compounds in open capillary tubes. ^1^H-NMR (DMSO-d_*6*_) and ^13^C-NMR (DMSO-d_*6*_) were recorded on Bruker Avance III 600 NMR spectrometer, ^1^H at 600 MHz and ^13^C at150 MHz using appropriate deuterated solvents. The results are conveyed in parts per million (*δ*, ppm) downfield from tetramethyl silane (internal standard). Proton NMR data are given as multiplicity (s, singlet; d, doublet; t, triplet; m, multiplet) and number of protons. Infrared (IR) spectra were recorded on a Bruker FTIR spectrometer. The mass spectral data was recorded on Waters Q-TOF micromass (ESI–MS). Elemental analysis for synthesized derivatives was performed on CHN analyzer (Additional files 1, 2 and 3).

### General procedure for the synthetic scheme 1

#### Step A: Synthesis of 4-(4-bromophenyl)-thiazol-2-amine (intermediate)

A mixture of *p*-bromo acetophenone (0.1 mol), thiourea (0.2 mol) and iodine (0.1 mol) was refluxed for 11–12 h. The reaction mixture was cooled and washed with diethyl ether to remove unreacted acetophenone and iodine. The completion of reaction was confirmed by thin layer chromatography. After this reaction mixture was allowed to cool and poured into the solution of ammonium hydroxide, precipitated and then filtered [26].

#### Step B: Synthesis of final derivatives (p1–p10)

A mixture of 4-(4-bromophenyl)-thiazol-2-amine (0.02 mol) and substituted aldehydes (0.02 mol) was refluxed in minimum amount of ethanol in presence of small amount of glacial acetic acid for 6–7 h. The completion of reaction was monitored by TLC. The mixture was cooled and poured in ice cold water. The solid thus obtained was filtered and dried [27].

### Antimicrobial evaluation (in vitro)

The antimicrobial activity of the synthesized molecules 4-(4-bromophenyl) thiazol-2-amine was evaluated against Gram positive bacteria [*Staphylococcus aureus* (MTCC3160) and *Bacillus subtilis* (MTCC441)], Gram negative bacterium *Escherichia coli* (MTCC443), and fungal strains—*Aspergillus niger* (MTCC281)*; Candida albicans* (MTCC227) by tube dilution method. The stock solution was prepared for the test compounds (**p1**–**p10**) and for the standard drugs (norfloxacin and fluconazole) in acetone to get a concentration of 100 μg/mL and this stock solution was further serially tube diluted [28]. Dilution of test and standard compounds were prepared with double strength nutrient broth-I.P (antibacterial) and sabouraud dextrose broth-I.P (antifungal) [29]. The samples were incubated at 37 ± 1 °C for 24 h (bacteria), 25 ± 1 °C for 7 days (*A. niger* and *C. albicans*), respectively and results were recorded in terms of MIC.

### Anticancer evaluation (in vitro)

The antiproliferative screening of synthesized 4-(4-bromophenyl)thiazol-2-amine molecules was conducted against the oestrogen receptor positive human breast adenocarcinoma, MCF7, in comparison to a standard drug (5-fluorouracil) using the SRB assay. Briefly, MCF7 cells were exposed to the compounds for 72 h. Treated cells were being fixed with trichloroacetic acid and then stained with 0.4% (*w/v*) SRB in 1% acetic acid. Unbound dye was removed by five washes with 1% acetic acid solution. Protein-bound dye was solubilized with 10 mM Tris base prior to reading of optical density using a computer-interfaced, 96-well microtiter plate reader. The anticancer activity result was expressed as mean IC_50_ value of at least triplicates [30].

### Molecular docking and ADME studies

#### Molecular docking

The selected target proteins (PDB ID-3ERT, 4WMZ and 1JIJ) required for molecular docking studies were obtained from the RCSB Protein data bank (http://www.rcsb.org/pdb/home/home.do) (Additional file 3). The selected PDB file was prepared for the molecular docking study using Protein Preparation Wizard (preprocessed, optimized and minimized). A grid is generated around the co crystallized ligand so that it can be excluded and new compounds can be attached to the same active site to study their interactions with receptor. The molecular structures of compounds that are to be docked must be in good representations of as they would appear in a protein–ligand complex. LigPrep module of Schrodinger *v11.5* was used to prepare the ligand (compound) for docking in Maestro format. The prepared ligand and receptor are docked using extra precision (XP). XP module docked the compounds with better precision and accuracy. The XP parameters like docking score glide energy and glide model value were calculated within the Schrodinger *v11.5* (Additional file 1) [17, 31–33].

#### ADME study

Most of the drug molecules fail during clinical trials, to streamline our study ADME properties determination is the crucial step. Drug likeliness and ADME properties of the most active compounds were determined using QikProp, GLIDE and Schrodinger *v11.5*. LigPrep module of Schrodinger *v11.5* was employed to prepare the ligand (compound) in Maestro format (*.maez*) for ADME study. Then we went on task, browsed the Qikpro dialogue box and ligand prepared file (*.maez*) of the synthesized derivatives was inserted to obtain the ADME parameters (Additional file 2) [34, 35].

## Conclusion

A novel series of thiazole derivatives was synthesized, docking study and evaluated in vitro antimicrobial and anticancer activities. Presence of electron releasing (OCH_3_, OH) and electron withdrawing (*p*-Br) groups at benzylidene ring of the compound **p2** made it as most potent antibacterial and anticancer agent. The presence of electron releasing group (OCH_3_) in compounds **p3** and **p4** also made them potent antifungal (*A. niger*) and antibacterial (*B. subtilis*) agents, respectively. The presence of fused aromatic nucleus in **p6** made it the most active antifungal agent against *C. albicans.* Molecular docking study of the selected most active compounds exhibited the best docked score and ADME properties with their better potency of antimicrobial and anticancer activities. The active compounds, **p2**, **p3**, **p4** and **p6** may act as useful leads for further development of antimicrobial and anticancer agents.

## Additional files

**Additional file 1.** Molecular Docking study of the synthesized compounds (**p1**–**p10**) and standard drugs.

**Additional file 2.** ADME properties of the most active synthesized compounds (**p2**–**p4** and **p6**).

**Additional file 3.** Proteins structures and PDB id link.

## Abbreviations

LBDD: ligand based drug designing
SBDD: structure based drug designing
ADME: absorption, distribution, metabolism and excretion
CADD: computer aided drug designing
ER-alpha: estrogen receptor alpha
NMR: nuclear magnetic resonance
IR: infrared
MCF7: Michigan Cancer Foundation7
SRB: Sulforhodamine B
MTCC: Microbial Type Culture Collection
μM: micro mole
PDB: protein data bank
XP: extra precision
5-Fu: 5-fluorouracil
H-bond: hydrogen-bond
2D: 2 dimensional
3D: 3 dimensional
SAR: structure activity relationship
MIC: minimum inhibitory concentration
RCSB: Research Collaboratory for Structural Bioinformatics
