# Supplementary material for: 4-(4-Bromophenyl)-thiazol-2-amine derivatives: synthesis, biological activity and molecular docking study with ADME profile
Source: BMC Chem. 2019 Apr 23;13(1):60. doi: 10.1186/s13065-019-0575-x (PMC6661755; doi:10.1186/s13065-019-0575-x)
Supplement: Supplementary file 1 — Additional file 1. Molecular Docking study of the synthesized compounds (p1–p10) and standard drugs. [file 13065_2019_575_MOESM1_ESM.pdf]

Additional File 1

Molecular Docking Results of the Synthesized Compounds and Standard Drugs

Antibacterial docking results

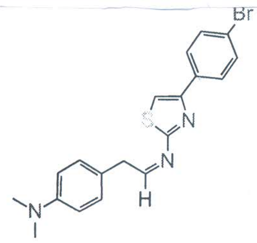

Comp. p3

title: 2nd.mol
glide energy: -51.681
entry name: 3rd.1
glide emodel: -72.266
docking score: -5.692

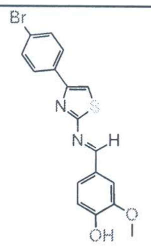

Comp. p2

title: ist.mol
glide energy: -49.479
entry name: 2nd.1
glide emodel: -72.456
docking score: -5.547

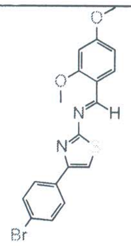

Comp. p8

title: 7th.mol
glide energy: -50.094
entry name: 8th.1
glide emodel: -60.355
docking score: -5.19

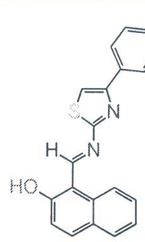

Comp. p6

title: 5th.mol
glide energy: -50.996
entry name: 6th.1
glide emodel: -72.659
docking score: -4.986

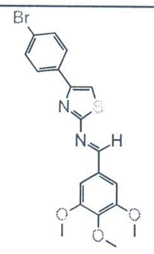

Comp. p4

title: 3rd.mol
glide energy: -54.654
entry name: 4th.1
glide emodel: -66.803
docking score: -4.845

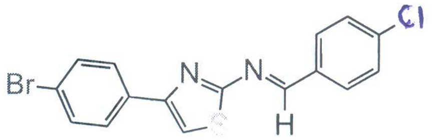

Comp. p7

title: 6th.mol
glide energy: -42.416
entry name: 7th.1
glide emodel: -53.65
docking score: -4.335

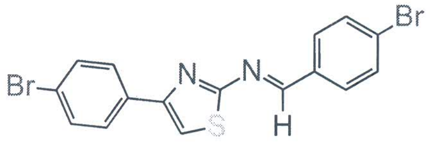

Comp. p5

title: 4th.mol
glide energy: -44.015
entry name: 5th.1
glide emodel: -55.619
docking score: -3.785

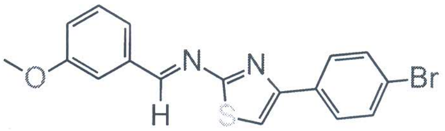

Comp. p9

title: 8th.mol
glide energy: -46.409
entry name: 9th.1
glide emodel: -59.529
docking score: -3.669

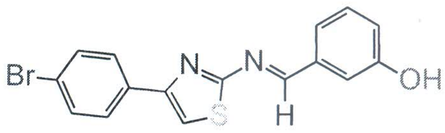

Comp. p10

title: 10th.mol
glide energy: -47.077
entry name: 10th.1
glide emodel: -63.222
docking score: -3.66

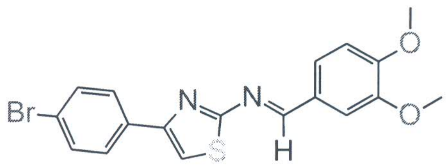

Comp. p1

title: Untitled Document-1
glide energy: -47.746
entry name: 1st.1
glide emodel: -60.919
docking score: -3.279

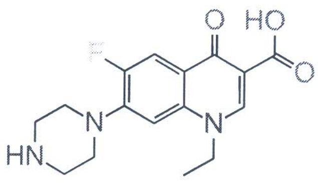

title: Untitled Document-1
glide energy: -53.349
entry name: NORFLOXacin.1
glide emodel: -73.93
docking score: -6.18

Antifungal docking results

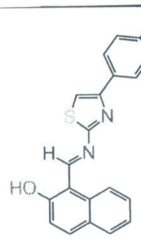

title: 5th.mol
glide energy: -45.842
entry name: 6th.1
glide emodel: -67.05
docking score: -8.342

Comp. p6

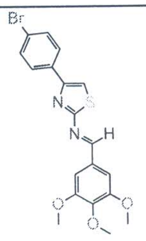

title: 3rd.mol
glide energy: -48.401
entry name: 4th.1
glide emodel: -62.199
docking score: -7.795

Comp. p4

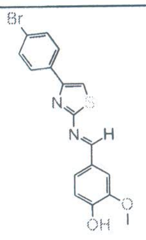

title: ist.mol
glide energy: -44.393
entry name: 2nd.1
glide emodel: -57.307
docking score: -7.792

Comp. p2

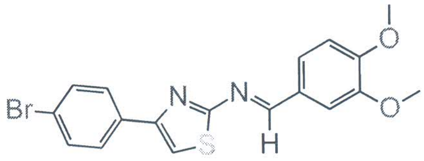

title: Untitled Document-1
glide energy: -48.336
entry name: 1st.1
glide emodel: -64.241
docking score: -7.547

Comp. p1

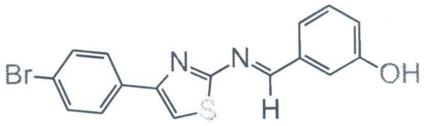

title: 10th.mol
glide energy: -39.006
entry name: 10th.1
glide emodel: -52.799
docking score: -7.49

Comp. p10

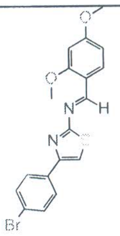

title: 7th.mol
glide energy: -41.737
entry name: 8th.1
glide emodel: -55.096
docking score: -7.231

Comp. p8

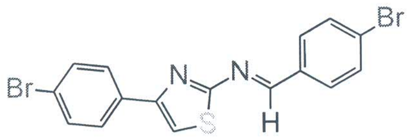

title: 4th.mol
glide energy: -41.869
entry name: 5th.1
glide emodel: -52.827
docking score: -6.883

Comp. p5

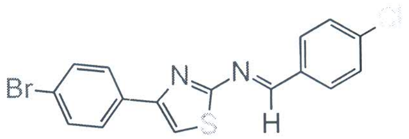

title: 6th.mol
glide energy: -41.853
entry name: 7th.1
glide emodel: -56.883
docking score: -6.802

Comp. p7

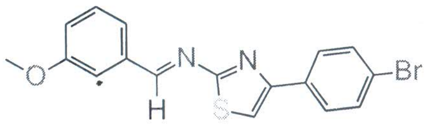

title: 8th.mol
glide energy: -46.656
entry name: 9th.1
glide emodel: -63.55
docking score: -6.687

Comp. p9

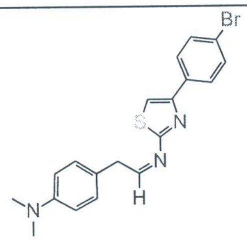

title: 2nd.mol
glide energy: -48.914
entry name: 3rd.1
glide emodel: -68.371
docking score: -6.513

Comp. p3

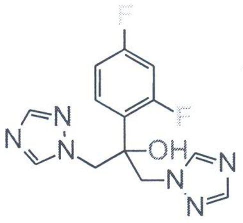

title: Untitled Document-1
glide energy: -40.932
entry name: fluconazole.1
glide emodel: -53.851
docking score: -5.587

<u>**Anticancer docking results**</u>

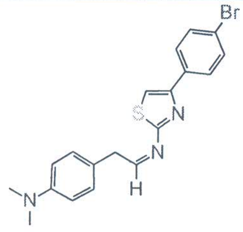

title: 2nd.mol
glide energy: -40.27
entry name: 3rd.1
docking score: -8.104
glide emodel: -58.326

Comp. p3

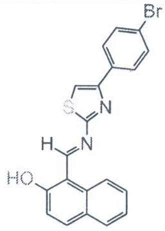

title: 5th.mol
glide energy: -44.9
entry name: 6th.1
docking score: -7.808
glide emodel: -61.547

Comp. p6

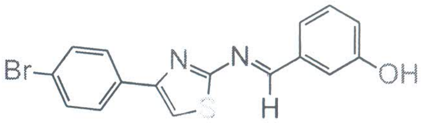

title: 10th.mol
glide energy: -40.163
entry name: 10th.1
docking score: -6.958
glide emodel: -55.096

Comp. p10

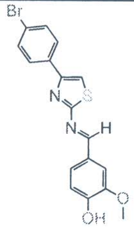

title: ist.mol
glide energy: -42.44
entry name: 2nd.1
docking score: -6.732
glide emodel: -55.844

Comp. p2

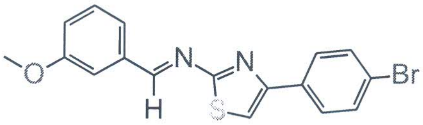

title: 8th.mol
glide energy: -41.113
entry name: 9th.1
docking score: -6.556
glide emodel: -55.141

Comp. p9

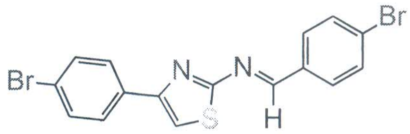

title: 4th.mol
glide energy: -41.329
entry name: 5th.1
docking score: -6.519
glide emodel: -50.478

Comp. p5

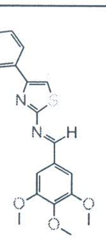

title: 3rd.mol
glide energy: -44.215
entry name: 4th.1
docking score: -6.191
glide emodel: -59.091

Comp. p4

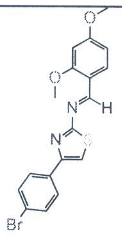

title: 7th.mol
glide energy: -34.63
entry name: 8th.1
docking score: -5.832
glide emodel: -50.092

Comp. p8

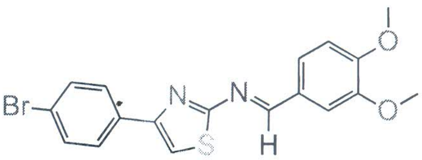

title: 9th mol
glide energy: -39.186
entry name: ist.1
docking score: -5.565
glide emodel: -49.291

Comp. p1

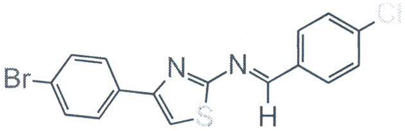

title: 6th.mol
glide energy: -38.286
entry name: 7th.1
docking score: -6.018
glide emodel: -50.627

Comp. p7
