# Supplementary material for: 4-(4-Bromophenyl)-thiazol-2-amine derivatives: synthesis, biological activity and molecular docking study with ADME profile
Source: BMC Chem. 2019 Apr 23;13(1):60. doi: 10.1186/s13065-019-0575-x (PMC6661755; doi:10.1186/s13065-019-0575-x)
Supplement: Supplementary file 2 — Additional file 2. ADME properties of the most active synthesized compounds (p2–p4 and p6). [file 13065_2019_575_MOESM2_ESM.pdf]

Additional File 2

ADME Study of the Most Active Compounds

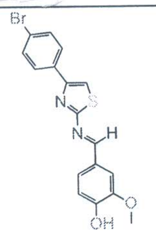

title: ist.mol
glob: 0.8009192
QPlogPo/w: 4.607
HumanOralAbsorption: 3
donorHB: 1.0
accptHB: 4.0
volume: 1056.326
PercentHumanOralAbsorption: 100.0
QPlogPw: 8.264
QPlogKp: -1.203
QPlogBB: -0.156
mol MW: 389.266
stars: None
source file index: 1
QPlogS: -5.98
RuleOfFive: 0

Comp. p2

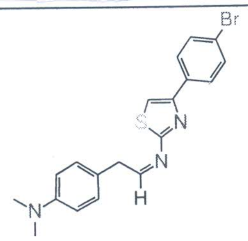

title: 2nd.mol
glob: 0.7968852
QPlogPo/w: 5.71
HumanOralAbsorption: 1
donorHB: 0.0
accptHB: 3.5
volume: 1158.251
PercentHumanOralAbsorption: 100.0
QPlogPw: 6.118
QPlogKp: -0.733
QPlogBB: 0.133
mol MW: 400.335
stars: None
source file index: 2
QPlogS: -6.28
RuleOfFive: 1

Comp. p3

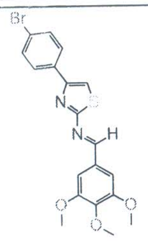

title: 3rd.mol
glob: 0.7935974
QPlogPo/w: 5.447
HumanOralAbsorption: 3
donorHB: 0.0
accptHB: 4.75
volume: 1191.069
PercentHumanOralAbsorption: 100.0
QPlogPw: 6.674
QPlogKp: -0.306
QPlogBB: 0.352
mol MW: 433.319
stars: None
source file index: 3
QPlogS: -6.144
RuleOfFive: 1

Comp. p4

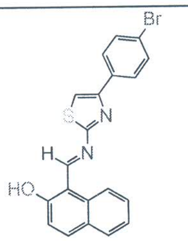

title: 5th.mol
glob: 0.8014526
QPlogPo/w: 5.52
HumanOralAbsorption: 1
donorHB: 1.0
accptHB: 3.25
volume: 1106.487
PercentHumanOralAbsorption: 100.0
QPlogPw: 8.608
QPlogKp: -0.477
QPlogBB: 0.09
mol MW: 409.299
stars: None
source file index: 4
QPlogS: -6.757
RuleOfFive: 1

Comp. p6
