# Supplementary material for: 4-(4-Bromophenyl)-thiazol-2-amine derivatives: synthesis, biological activity and molecular docking study with ADME profile
Source: BMC Chem. 2019 Apr 23;13(1):60. doi: 10.1186/s13065-019-0575-x (PMC6661755; doi:10.1186/s13065-019-0575-x)
Supplement: Supplementary file 3 — Additional file 3. Proteins structures and PDB id link. [file 13065_2019_575_MOESM3_ESM.docx]

**Additional File 3**

Protein structures of the selected PDB ID and web link: <http://www.rcsb.org/pdb/home/home.do>

**
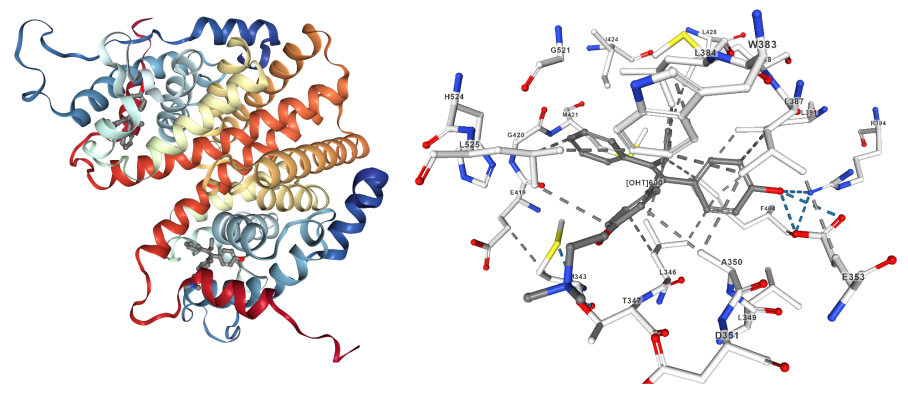
**

**PDB ID: 3ERT for the most active anticancer compounds**


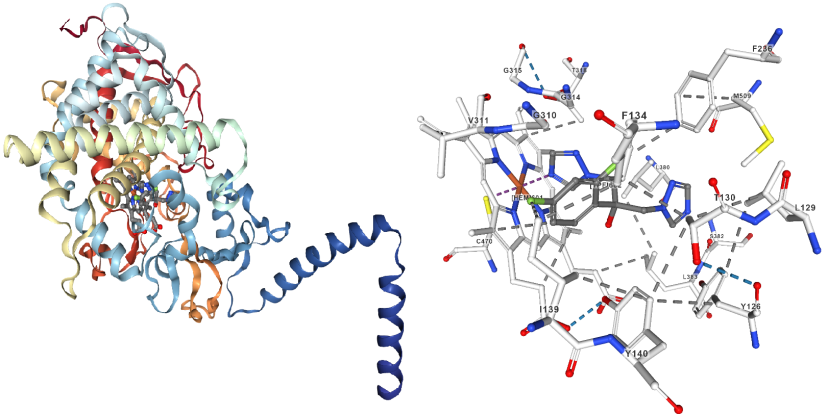


**PDB ID: 4WMZ for the most active antifungal compounds**

**
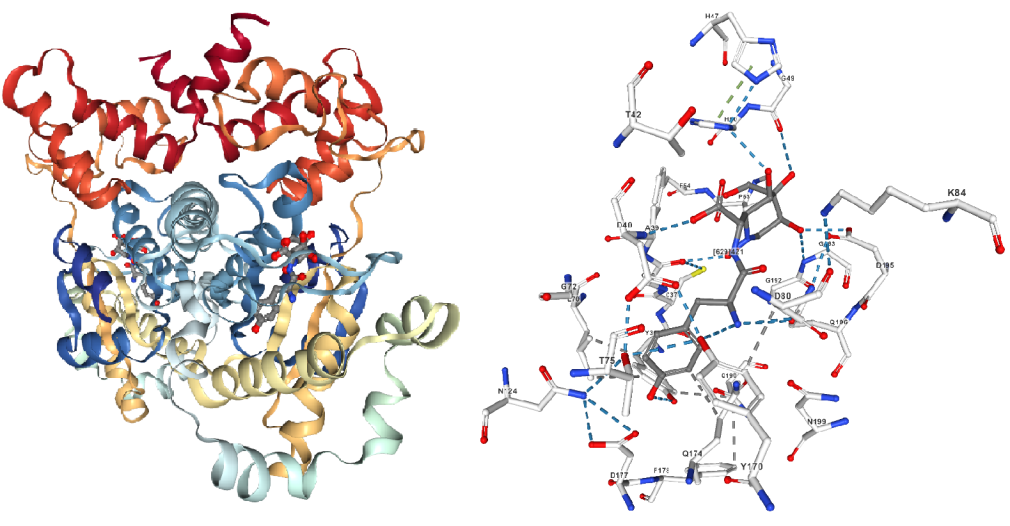
**

**PDB ID: 1JIJ used for the most active antibacterial compounds**
